# Disruption of the *Sec24d* Gene Results in Early Embryonic Lethality in the Mouse

**DOI:** 10.1371/journal.pone.0061114

**Published:** 2013-04-15

**Authors:** Andrea C. Baines, Elizabeth J. Adams, Bin Zhang, David Ginsburg

**Affiliations:** 1 Johns Hopkins Bayview Internal Medicine Residency Program, The Johns Hopkins University, Baltimore, Maryland, United States of America; 2 Life Sciences Institute, University of Michigan, Ann Arbor, Michigan, United States of America; 3 Genomic Medicine Institute, Lerner Research Institute, Cleveland Clinic Foundation, Cleveland, Ohio, United States of America; 4 Departments of Internal Medicine, Human Genetics, and Pediatrics and the Howard Hughes Medical Institute, University of Michigan, Ann Arbor, Michigan, United States of America; Institute of Molecular and Cell Biology, Singapore

## Abstract

Transport of newly synthesized proteins from the endoplasmic reticulum (ER) to the Golgi is mediated by the coat protein complex COPII. The inner coat of COPII is assembled from heterodimers of SEC23 and SEC24. Though mice with mutations in one of the four *Sec24* paralogs, *Sec24b*, exhibit a neural tube closure defect, deficiency in humans or mice has not yet been described for any of the other *Sec24* paralogs. We now report characterization of mice with targeted disruption of *Sec24d*. Early embryonic lethality is observed in mice completely deficient in SEC24D, while a hypomorphic *Sec24d* allele permits survival to mid-embryogenesis. Mice haploinsufficient for *Sec24d* exhibit no phenotypic abnormality. A BAC transgene containing *Sec24d* rescues the embryonic lethality observed in *Sec24d*-null mice. These results demonstrate an absolute requirement for SEC24D expression in early mammalian development that is not compensated by the other three *Sec24* paralogs. The early embryonic lethality resulting from loss of SEC24D in mice contrasts with the previously reported mild skeletal phenotype of SEC24D deficiency in zebrafish and restricted neural tube phenotype of SEC24B deficiency in mice. Taken together, these observations suggest that the multiple *Sec24* paralogs have developed distinct functions over the course of vertebrate evolution.

## Introduction

Approximately one-third of all vertebrate proteins traverse the intracellular secretory pathway prior to being secreted into the extracellular space or transported to any of a number of intracellular compartments, including the Golgi, endosome, lysosome, or plasma membrane [1], [2], [3]. Following co-translational translocation into the endoplasmic reticulum (ER) lumen, newly synthesized proteins are folded and undergo initial post-translational modification, followed by exit from the ER at ribosome-free regions called ER exit sites (ERES) [4] via COPII-coated vesicles [1], [5], [6]. In yeast, the COPII coat is composed of the small GTP-binding protein Sar1p, the heterodimeric Sec23p/Sec24p complex and the heterotetrameric Sec13p/Sec31p complex [7]. Sar1p generates membrane curvature and initiates vesicle formation by inserting an N-terminal amphipathic helix into the ER membrane [8]. The active membrane-bound Sar1p-GTP recruits Sec23p/Sec24p, and Sec24p drives the selective recruitment of cargo proteins into budding vesicles [9], [10], [11]. Polymerization of the outer Sec13p/Sec31p complex is the final step in vesicle budding [12].

While the components of the COPII coat are highly conserved, and the fundamental interactions appear to be similar from yeast to mammals, most components exhibit multiple paralogs in higher eukaryotes. Studies in yeast suggest that Sec24p is the major cargo binding component of the COPII coat, with three cargo-binding sites in the N-terminal region interacting with either cytoplasmic domains of the cargo itself or cargo adaptors [9]. Deletion of yeast Sec24p is lethal, whereas deletion of either of two non-essential Sec24p paralogs, Lst1p and Iss1p, results in specific cargo-transport defects [13], [14], [15]. In vertebrates, four *Sec24* paralogs (*SEC24A-D*) have been identified. These *Sec24* paralogs fall into two subfamilies, a SEC24A/B subgroup and a SEC24C/D subgroup, based on protein sequence similarity, with the A/B subgroup closer to yeast Sec24p [16]. All four paralogs contain highly conserved C-terminal domains and a more variable N-terminal region, while the SEC24A/B and SEC24C/D subgroups appear to differ in their affinity for a subset of known cargo-sorting signals [17], [18].

Mice with mutations in *Sec24b* exhibit neural tube closure defects as a result of decreased VANGL2 trafficking out of the ER [19], though no human disorders resulting from deficiencies of SEC24B or any of the other *Sec24* paralogs have been reported. Mutations in the SEC24 binding partner SEC23, which has two paralogs (*Sec23a* and *Sec23b*), have been characterized both in humans and in fish. Missense mutations in human *SEC23A* lead to cranio-lenticulo-sutural dysplasia (CLSD), characterized by the persistence of wide-open fontanelles into childhood and the development of Y-shaped cataracts [20]. Mutations in human *SEC23B* cause congenital dyserythropoietic anemia type II (CDAII), characterized by a specific defect in erythrocyte development [21] while *Sec23b* deficient mice have a markedly different phenotype, exhibiting pancreatic disruption and disintegration [22]. Disruption of either *Sec23a or Sec23b* in zebrafish both result in defects in extracellular matrix (ECM) protein secretion, producing a phenotype reminiscent of CLSD in humans [20], [23]. Zebrafish lacking SEC24D exhibit similar craniofacial dysmorphology, presumably due to defects in the trafficking of extracellular matrix (ECM) proteins including type II collagen and matrilin [24] and medaka fish with a nonsense mutation in *sec24d* have also have skeletal defects [25]. We now report the characterization of murine SEC24D deficiency. Mice null for SEC24D exhibit very early embryonic lethality, suggesting an essential role for SEC24D in the transport of critical protein cargoes from the ER.

## Materials and Methods

### Ethics Statement

All animal care and use complied with the Principles of Laboratory and Animal Care established by the National Society for Medical Research. The University of Michigan’s University Committee on Use and Care of Animals (UCUCA) approved all animal protocols in this study under protocol number 08571.

### Generation of SEC24D Deficient Mice

ES cell clones RRT226 and RRR785 were obtained from the International Gene Trap Consortium (IGTC, Bay Genomics, San Francisco, CA), and will be referred to as Sec24d^gt^ and Sec24d^gt2^, respectively. Both ES cell clones were cultured as described [26] and expanded for microinjection and preparation of total RNA and genomic DNA. ES cell-mouse chimeras were prepared by blastocyst microinjection as described [27] and bred with C57BL/6J mice to obtain germ-line transmission. ES-cell derived F1 agouti offspring were genotyped using primers Neo A and Neo B to amplify a region of the neomycin cassette to determine the presence (Neo+) or absence (Neo-) of the gene trap allele. Sequences for all primers used in this study are listed in Table 1. Mice carrying the gene trap allele were maintained by backcrossing to C57BL/6J.

**Table 1:** Primer sequences used in this study (denoted 5′ to 3′).

Mapping and Genotyping Primers		Primers for BAC Transgene Rescue			
In8F1	CCATGCAGTGCTACACAAGC	pBACe3.6F1	GCTGCAGATCCCTAAACAGC		
In8F2	CTGCTGCCTGAAGATCAAGA	pBACe3.6R1	TTCCGTCTCCGTCAAAAATC		
In8F3	CATTCGTTGCTCCTCCTCTT	MS-1F	TGGTAGCAGGACACAGCTGATA		
In8F4	AATCTGACCTGGGGAGAAGC	MS-1R	GGTCTAACACACGAGAATTTGAA		
In8F5	CCCAACCCTCACGACAATAA	MS-2F	GCATGGAAAAACCCTGTCTC		
In8F6	GCAGAGGCTGCTATTCCATC	MS-2R	CACCATTCAGCAATGATTCTC		
In8F7	ATGGATGCTGCTGGAACTCT	MS-3F	TTCGGCTATTGTCTTCCACA		
In8F8	CACAGGGAAAACGTGGAAAG	MS-3R	ACGGGGTTAGGTAGCCAGAT		
In8F9	CGTGTCCTTCCCTAAACAGC	MS-4F	TGAGTCTGGCTAATTGCACTG		
In8F10	CAGGTGGGGGATCTTATGAG	MS-4R	GATGGGAGGAGCATTCTGAG		
In8F11	GGTGCTTTCAAATTGGTCAC	**Southern Blot Probe Primers**			
In8F12	GATGGCGTGTAAGCTGTTGA				
In8F13	GGGACAAAACAGCAGCCTAC	ApaI A		AATCCGTGGTTGTAGGTTGC	
In8F14	CACTGGGGATATGGAACCTG	ApaI B		CAAAGGATCTCCCCACTCTG	
In8F15	GGTGGGGAAGAGAACTTGTG	24dEx20-21ProbeF		ACAGTTTGTTGAAAAACTG	
In8F16	CTGGCCTCTTTACACCCTTG	24dEx20-21ProbeR		ACGTGTGGATTGGCAGGAGCAG	
In8F17	AAAGAGCGAGACCAACCTGA	**RT-PCR Primers**			
In8F18	TTTTCCTGTAGGCCCATGAC				
In8F19	AGCACAGGGAAGCCTAAGTG	GAPDH-F			TGTGATGGGTGTGAACCACGAGAA
In8F20	CCCTTTCCTCTTCCTCCACT	GAPDH-R			ACCAGTGGATGCAGGGATGATGTT
In8F21	GAGGTCAGAAGAGGGATCA	Sec24dEx20-21F			TGAAGGTGCTGCCTGTGTACATGA
In20F1	GGCAGTGGAAGGTGTAAGGA	Sec24dEx20-21R			ACATGTTCAGACCGTTAGCCAGCA
In20F2	GCCATGCAAGAGTCCCTCAGT				
In20F3	GCCCCTGTCTCTAAGCCTCT				
In20F4	CATCCTGTTCGTCCTCCATC				
In20F5	TGATCGGTTGCCACATAAAA				
In20F6	CCCTAGTCGGGCTCTTACCT				
In20F7	GGCCTTTCTCCCTCAAAAAG				
In8R4	TGTCCAGGAAACACGACAGA				
In20R1	CTGGCCCTGAATTTATTGTGTG				
Vector 19	GGGTCTCAAAGTCAGGGTCA				
Vector 20	GACCTGGCTCCTATGGGATA				
NeoA	CTTGCGCAGCTGTGCTCGACGTTG				
NeoB	TCTTCGTCCAGATCATCCTGATCG				

### Mapping of the Gene Trap Vector Insertion Sites

The gene trap vector insertion sites in intron 8 of Sec24d^gt^ and intron 20 of Sec24d^gt2^ were determined by PCR amplification and DNA sequencing. A series of forward primers evenly spaced throughout the intronic sequence (I8F1-21 and I20F1-7, Table 1) were combined with a reverse primer (Vector 19 or Vector 20, Table 1) specific to the 5′end of the gene trap vector sequence. Amplicons corresponding to a specific product spanning the insertion site were confirmed by DNA sequencing. Insertion site sequences for both gene trap alleles were deposited into GenBank.

### Genotyping Mice by PCR and Southern Blot

**Figure 1:**
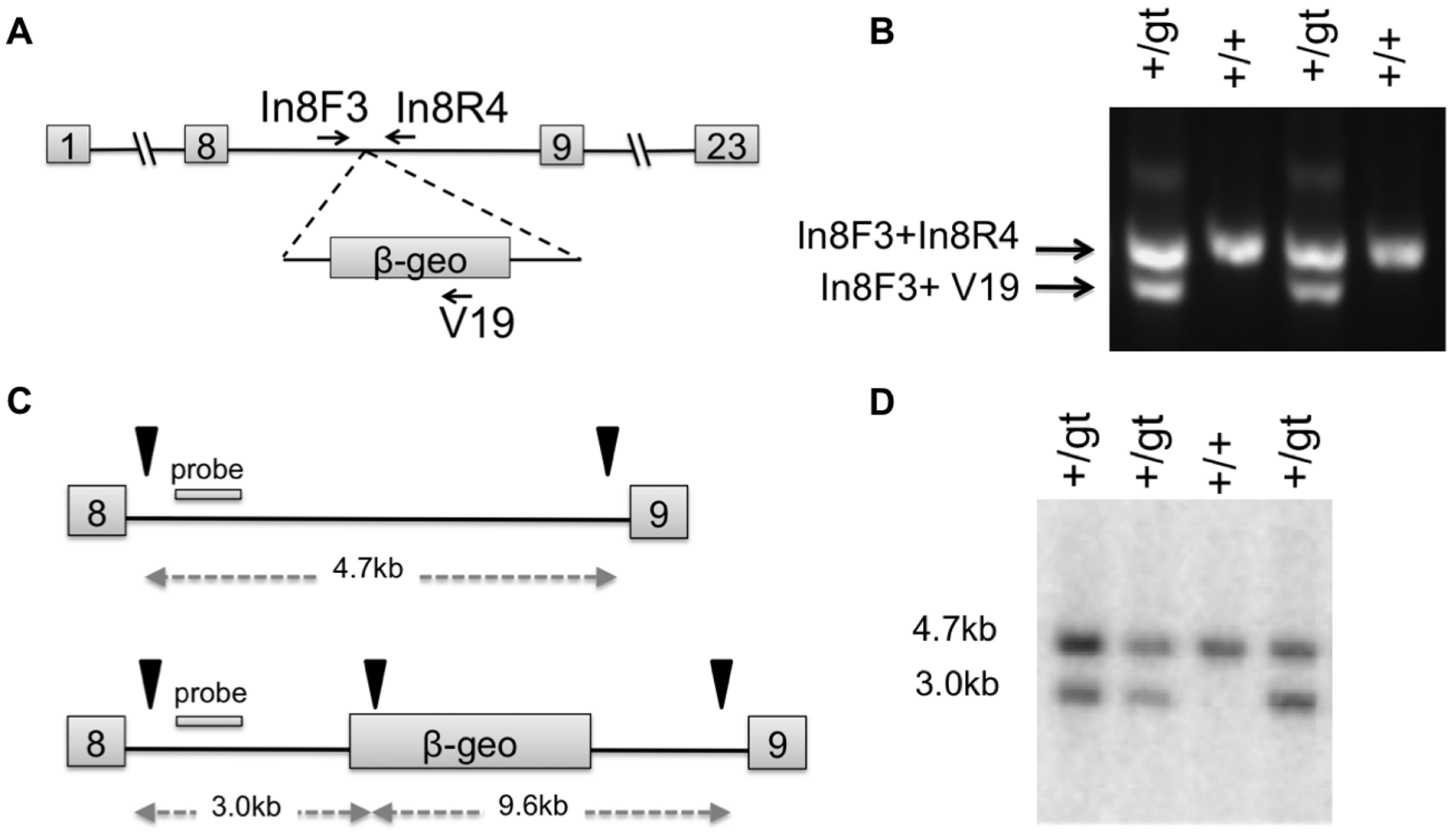
Generation of SEC24D deficient mice. (A) Schematic representation of the Sec24d^gt^ gene trap allele in intron 8 at the mouse *Sec24d* locus. (B) Genotype results using a three-primer competitive PCR assay with the primers indicated in A. (C-D) Confirmation of the gene trap insertion by Southern blot analysis of ApaI-digested Sec24d^gt^ genomic DNA from *Sec24d^+/+^* and *Sec24d^+/gt^* mice; ApaI restriction enzyme sites (arrows) and probe location are depicted in C. Hybridization to the wild-type allele detected a 4.7 kb ApaI restriction fragment, whereas hybridization to the gene trap allele detected a 3.0 kb ApaI restriction fragment. In mice heterozygous for the Sec24d^gt^ gene trap allele, both restriction fragments are present.

**Figure 2:**
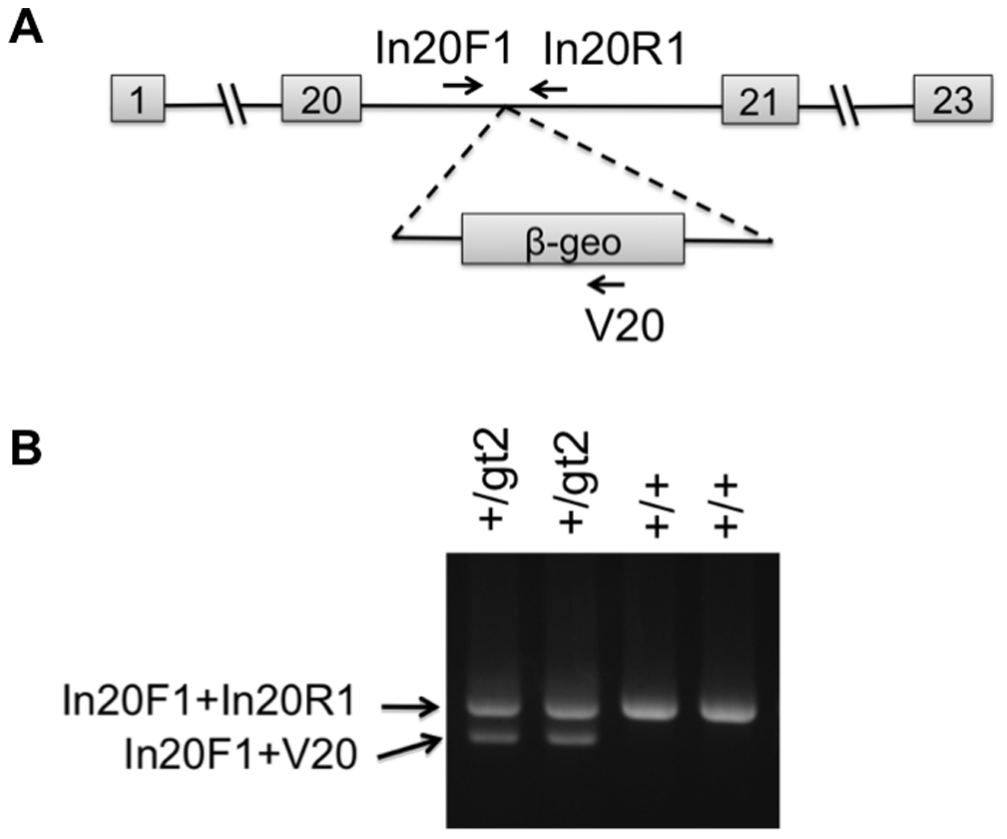
Generation of a second allele for SEC24D deficient mice. (A) Schematic representation of the Sec24d^gt2^ gene trap allele in intron 20 at the mouse *Sec24d* locus. (B) A three-primer competitive PCR genotyping assay to identify the *Sec24d^gt2^* allele. Primer locations are depicted in (A).

Mice from Sec24d^gt^ were genotyped using a three-primer competitive PCR assay consisting of a common forward primer, (In8F3) located upstream of the insertion site in intron 8, and two reverse primers, located downstream of the insertion site in intron 8, (In8R4) or within the gene trap vector (V19) (Figure 1A). This reaction produces products of different sizes from the wild-type (762 bp) and gene trap (666 bp) alleles, which are resolved by agarose gel electrophoresis (Figure 1B). Genotypes for four representative Sec24d^gt^ mice were also confirmed by Southern blot analysis using a 371 bp probe amplified from C57BL/6J genomic DNA with the primers ApaI A and ApaI B. The probe was hybridized to ApaI-digested genomic DNA, as previously described [28]. Mice from Sec24d^gt2^ were also genotyped using a three-primer competitive PCR, with a common forward primer (In20F1) located upstream of the insertion site in intron 20, and two reverse primers, located downstream of the insertion site (In20R1) in intron 20 or within the gene trap vector (V20) (Figure 2A). This reaction produces a 715 bp product from the wild-type allele and a 558 bp product from the gene trap allele, which are resolved by agarose gel electrophoresis (Figure 2B).

### Timed Mating

Timed matings were performed by intercrossing *Sec24d* heterozygous mice. Embryos were harvested at multiple time points, including day E10.5–11.5 for genotyping and histological analysis and E13.5 for the preparation of mouse embryonic fibroblasts. Genotyping was performed on genomic DNA isolated from embryonic yolk sacs. For blastocyst collection, female *Sec24d^+/gt^* or *Sec24d^+/gt^* ^2^ mice were superovulated by intraperitoneal injection of 0.5 IU pregnant mares’ serum gonadotropin (PMSG) on day 1 and 0.5 IU human chorionic gonadotropin HCG on day 3. Females were then mated with *Sec24d^+/gt^* or *Sec24d^+/gt^* ^2^ males, and copulation plugs were noted on day 4, counted as day E0.5 of embryonic development. Blastocysts were harvested on day 7 (E3.5) as previously described [29], and crude lysates were genotyped by three-primer PCR. Morula (8-cell embryos) were collected from juvenile superovulated *Sec24d^+/gt^* female mice mated with *Sec24d^+/gt^* male mice and placed in culture as described [30].

### Reverse-transcription PCR

Total RNA was isolated from a panel of frozen tissues from wild-type mice, *Sec24d^+/gt^* mice and wild-type E10.5 embryos using the RNeasy Mini Kit (Qiagen), as per manufacture’s instructions, including the optional DNaseI digestion step. cDNA synthesis and PCR were carried out in one reaction using the SuperScript® III One-Step RT-PCR System with Platinum®Taq (Invitrogen). Primers were designed such that amplicons for each gene were approximately the same size. Primer sequences are listed in Table 1.

### Quantitative Analysis by Southern Blot

To determine the limit of sensitivity for our PCR assay, serial dilutions of total RNA from wild-type into total RNA from *Sec24d^gt2/gt2^* embryos were used as template for RT-PCR. PCR products 190 bp in length were amplified from the resulting cDNA using Sec24dExon20-21F and Sec24dExon20-21R (Table 1) and analyzed by Southern blotting using a 144 bp ^32^P-labelled DNA probe generated from wild-type cDNA using primers 24dEx20-21ProbeF and 24dEx20-21ProbeR (Table 1).

### Generation of BAC Transgenic Mice

Two bacterial artificial chromosome (BAC) clones containing the entire *Sec24d* gene, RP23-355K12 (RP23) and RP24-271N12 (RP24) were obtained from the BACPAC Resources Center at Children’s Hospital Oakland Research Institute (CHORI, http://bacpac.chori.org/). BAC DNA was purified using a NucleoBond^®^ BAC 100 kit (Machery-Nagel), per manufacturer’s instructions. C57BL/6J×SJL F1 female mice were generated by the University of Michigan Transgenic Animal Model Core and crossed to *Sec24d* heterozygous mice. Zygotes from this cross were injected with BAC DNA and transgenic founders for RP23 and RP24 were detected by PCR using pBACe3.6F1 and pBACe3.6R1 (Table 1), primers specific for the vector backbone.

Transgenic founders carrying the BAC transgene (*Tg+*) were generated using *Sec24d^+/gt^* females as the egg donors, resulting in both *Sec24d^+/+^* and *Sec24d^+/gt^* founders for RP23 or RP24. *Sec24d^+/gt^ Tg+* founders were immediately crossed with mice heterozygous for the *Sec24d* gene trap allele (*Sec24d* ^+/*gt*^ *)* to generate potential *Sec24d^gt/gt^ Tg+* rescues. To generate *Sec24d^+/gt^ Tg+* for lines with wild-type founders, an additional cross between *Sec24d^+/+^ Tg+* and mice heterozygous for the *Sec24d* gene trap allele (*Sec24d* ^+/*gt*^ *)* was required. The resulting *Sec24d^+/gt^ Tg+* mice were crossed with *Sec24d^+/gt^* mice to generate potential *Sec24d^gt/gt^ Tg+* mice. All progeny were subjected to genotyping for *Sec24d* as well as the presence of the BAC transgene. However, these assays cannot distinguish the endogenous wild-type allele from the copy of *Sec24d* present on the BAC-transgene. Thus, *Sec24d^gt/gt^ Tg+* mice were distinguished from *Sec24d^+/gt^ Tg+* mice by genotyping for microsatellites differing between the *Sec24d* “+” and “gt” alleles (see below). All *Sec24d^+/gt^* mice used for this study had been backcrossed to C57BL/6J (≥N8).

### Microsatellite Genotyping

**Figure 3:**
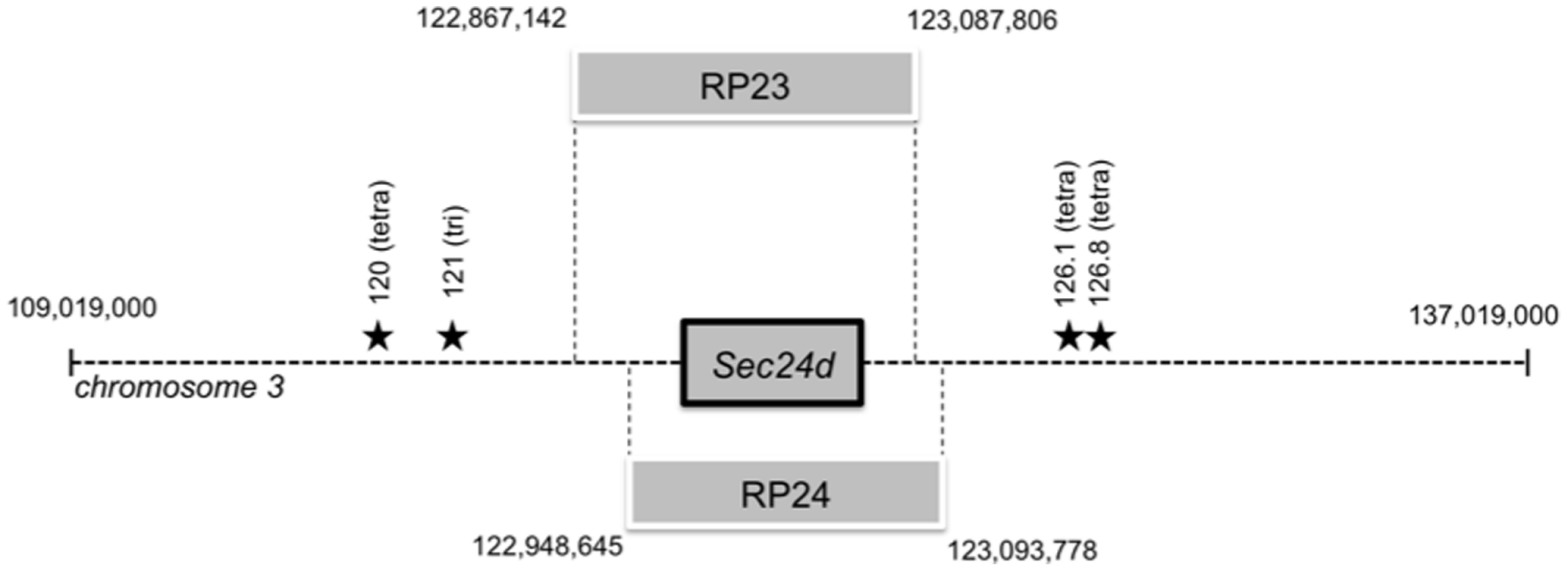
Locations of BACs and microsatellite markers on mouse chromosome 3. BAC transgenes RP23-355K12 (RP23) and RP24-271N12 (RP24) contain the entire *Sec24d* gene as well as upstream and downstream sequences as indicated. Stars depict relative locations of the four microsatellite markers used for microsatellite genotyping. Exact genomic locations are listed for genotyping primers are listed in Table 1.

A microsatellite genotyping assay was designed to distinguish the wild-type allele from the gene trap allele originally targeted on the 129S1/SvImJ background. To ensure that the correct genotype assignments were given, four independent microsatellites near *Sec24d* but outside both BAC transgenes were chosen, two on either side of *Sec24d* (Figure 3). These microsatellites, three tetra-nucleotide repeats and one tri-nucleotide repeat, were selected for use in the microsatellite genotyping assay using the Tandem Repeat Database [31] because they differed in allele size among the relevant mouse strains to distinguish the endogenous *Sec24d* from the copy of the *Sec24d* gene in the BAC-transgene. Each microsatellite was evaluated for every potentially transgenic *Sec24d^gt/gt^* mouse by PCR on genomic DNA using GoTaq^®^ Hot Start Green Master Mix (Promega). A forward primer located upstream and a reverse primer located downstream of the microsatellite repeat were used for amplification (see primer sequences, Table 1). Primers were designed using Primer3 such that the amplicon size was approximately 200 bp in length, based on the C57BL/6J reference sequence. PCR was performed as per manufacturer’s instructions, using 29 cycles and scaled up to a 30 µl reaction volume. Amplification annealing temperatures were optimized for each primer set. PCR products were separated by PAGE using 20% polyacrylamide gels and ethidium bromide staining. The gene trap allele is expected to be 129/SvImJ within the congenic interval, in contrast to wild-type alleles, which should be either C57BL/6J, DBA/2J, or SJL/J, based on the breeding strategy. (SJL/J was introduced with some of the original transgenic founders, and DBA/2J with some early matings, though all subsequent backcrosses were to C57BL/6J). Genomic DNA isolated from pure C57BL/6J, DBA/2J, 129S1/SvImJ, and SJL/J mouse strains was also used as templates to determine the amplicon size corresponding to each strain for each microsatellite marker. The genotypes of mice identified as *Sec24d^gt/gt^ Tg+* were confirmed by progeny testing through crosses with *Sec24d^+/gt^* mice (≥ N7 on C57BL6/J). Data shown in the tables excludes 6 mice in which a recombination event occurred between the upstream and downstream sets of markers. This number is consistent with the predicted recombination frequency of ∼1∶50 within this 4.2 Mb interval. We cannot exclude the possibility that we missed a double recombination event, though the chance of that occurring within our sample size is unlikely (predicted frequency for double recombinants, ∼1∶2500).

### Statistical Analysis

To determine statistical deviation from the expected Mendelian ratios of genotypes from a given cross, the *p*-value reported is the chi-squared value of observed ratio of genotypes compared to the expected ratio. Complete blood counts parameters were evaluated for significance using Student’s T-test comparing levels from wild-type mice to levels from *Sec24d^+/gt^* mice. An initial analysis showed no significant difference between males and females for each genotype, therefore data from males and females were pooled. The wild-type group consisted of 1 male and 2 females, and the *Sec24d^+/gt^* group consisted of 2 males and 2 females. Alpha levels were adjusted for multiple observations according to the Bonferonni correction.

## Results

### SEC24D is Required for Early Embryonic Development in the Mouse

**Figure 4:**
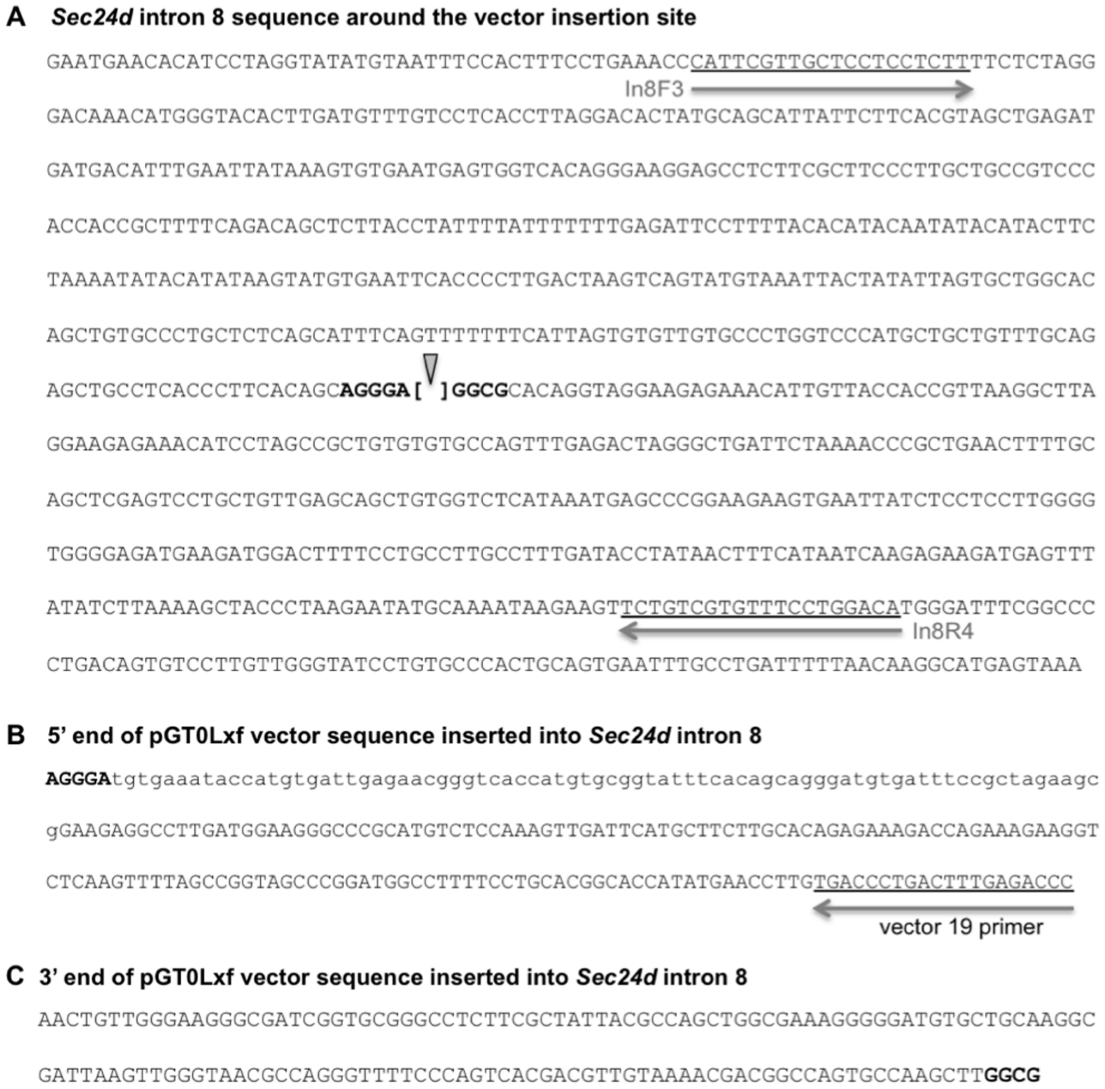
Sec24d^gt^ allele sequence. (A) Sequence of the Sec24d^gt^ gene trap insertion site in intron 8 of the *Sec24d* gene. The arrowhead indicates the site of the gene trap vector pGT0LxF insertion, with the flanking *Sec24d* intron 8 sequence in bold. The locations of genotyping primer sequences are underlined. (B) 5′ end of the vector sequence inserted into intron 8. Sequence aligning to intron 8 is in bold, while the lowercase sequence represents a 77 bp insertion that is not present the pGT0L×F vector and cannot be identified in the mouse genome. The position of primer V19 within the 5′ vector sequence is indicated. (C) 3′ end of the vector sequence inserted into intron 8. Intron 8 sequence is in bold.

Genomic PCR and sequencing identified the Sec24d^gt^ gene trap insertion site at position 3378 of intron 8, numbering from the start of the intron (GenBank accession number KC763189) (Figures 1A,B, 4A–C). This insertion is consistent with the mRNA RT-PCR data mapping the gene trap to exon 8 [32] and is anticipated to disrupt SEC24D, generating a fusion transcript containing SEC24D exons 1 through 8 (encoding amino acids 1–347 of the total 1032 in SEC24D) fused to the β-geo selection cassette. Germline transmission of the *Sec24d^gt^* allele was achieved, as confirmed by PCR and Southern blot analysis (Figure 1B–D).

The genotypes of progeny mice generated from *Sec24d^+/gt^* intercrosses are shown in Table 2. Of 209 pups genotyped at weaning, no *Sec24d^gt/gt^* mice were observed (*p*<7×10^−17^). Similarly, 0/28 and 0/27 *Sec24d^gt/gt^* genotypes were observed at E10.5–11.5 or the blastocyst stage, respectively. Only 1 out of 17 embryos genotyped at the 8-cell stage was *Sec24d^gt/gt^* (*p*<0.07). Though a statistically significant excess of *Sec24d^+/gt^* mice was observed (*p*<0.001) in intercross progeny at all of the above stages, analysis of a much larger number of backcross progeny (634) was consistent with the expected Mendelian ratio of 50∶50 for wild-type and heterozygous mice (p>0.8).

**Table 2:** Results of *Sec24d^+/gt^* intercrosses, and backcrosses to *Sec24d^+/+^* mice.

*Sec24d* genotype:	+/+	+/gt	gt/gt	*p-*value
***Sec24d^+/gt^*** ** × ** ***Sec24d^+/gt^*** ** expected:**	**25%**	**50%**	**25%**	
Progeny at weaning (n = 209)	21.1% (44)	78.9% (165)	0	<7.1×10^−17^
E10.5 to E11.5 (n = 28)	21.4% (6)	78.6% (22)	0	<2.3×10^−3^
Blastocyst (n = 27)	29.6% (8)	70.4% (19)	0	<2.7×10^−3^
8-cell embryo (n = 17)	11.8% (2)	82.4% (14)	5.8% (1)	<6.0×10^−2^
***Sec24d^+/gt^*** ** × ** ***Sec24d^+/+^*** ** expected:**	**50%**	**50%**	**–**	
N2 to N17 Progeny at weaning (n = 634)	49.5% (314)	50.5% (320)	–	>0.81

### No Phenotypic Abnormalities in *Sec24d^+/gt^* Mice

**Figure 5:**
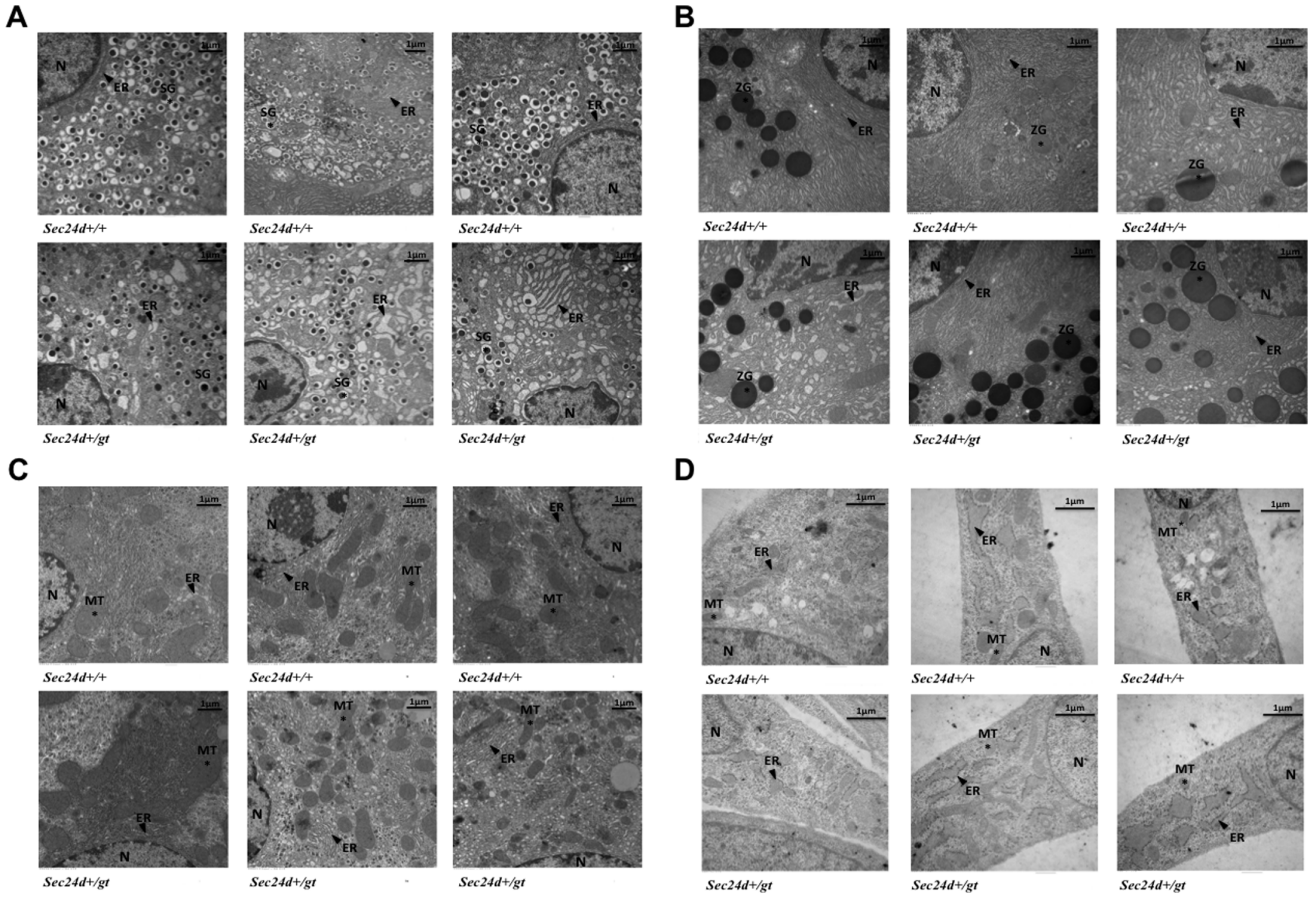
Transmission electron micrograph of *Sec24d^+/+^* and *Sec24d^+/gt^* cells. (A) pancreatic islet cells, (B) pancreatic acinar cells, (C) hepatocytes, and (D) mouse embryonic fibroblasts. Islet cells are identified by the presence of specialized secretory granules and acinar cells by the presence of zymogen granules. Samples were viewed at 10,500–13,500× direct magnification. A scale bar at the top right of each image corresponds to 1 µm. Abbreviations: N = nucleus, ER = endoplasmic reticulum (black arrowheads), SG = secretory granules, ZG = zymogen granules, MT = mitochondria.

*Sec24d^+/gt^* mice are viable and fertile and exhibit no gross or microscopic abnormalities on standard autopsy examination. Complete blood count analysis identified no significant differences between *Sec24d^+/gt^* and *Sec24d^+/+^* littermates for most parameters measured after correction for multiple observations (Table 3). Electron microscopy of pancreas and liver tissues from *Sec24d^+/gt^* mice, as well as mouse embryonic fibroblasts derived from *Sec24d^+/gt^* mice, showed no abnormalities in the cellular organization or ER structure compared to tissues or fibroblasts derived from littermate *Sec24d^+/+^* controls (Figure 5A–D).

**Table 3:** Complete blood count analysis of *Sec24d^+/+^* and *Sec24d^+/gt^* mice.

Sec24d^+/+^ Sec24d^+/gt^ p-value*			
**WBC (× 10^3^ cells/μl)**	5.50±0.74	7.25±0.63	>0.12
**RBC (× 10^6^ cells/μl)**	9.57±0.22	9.93±0.13	>0.19
**HGB (g/dL)**	15.67±0.88	16.0±0.0	>0.67
**HCT (%)**	45.0±1.0	46.25±0.75	>0.35
**MCV (fL)**	46.93±0.35	47.35±0.23	>0.34
**MCH (pg)**	16.10±0.74	16.15±0.06	>0.93
**MCHC (g/dL)**	34.37±1.68	34.08±0.13	>0.84
**RDW (%)**	12.07±0.09	11.75±0.09	>0.04
**PLT (× 10^3^ cells/μl)**	950.0±120.1	1035.0±46.3	>0.49
**MPV (fL)**	6.13±0.52	6.50±0.17	>0.48

Whole blood was drawn by retro-orbital puncture and analyzed on an Advia120 whole blood analyzer. All values are+or – standard error of the mean. *Based on the Bonferonni correction for multiple observations, the level of significance corresponding to *p<*0.05 for a single observation would be adjusted to *p*<3.8×10^−3^.

### *Sec24d* is Ubiquitously Expressed

Analysis of *Sec24a-d* mRNA expression by real-time RT-PCR detected expression of all four *Sec24* paralogs at E10.5, E14, and E18.5, consistent with data from the EMAGE gene expression database (http://www.emouseatlas.org/emage/) [33]. RT-PCR analysis of *Sec24d* expression in adult mouse tissues demonstrated broad expression in a wide range of tissues, similar to previous reports of human expression patterns [16] from the RNA Atlas [34]. The latter identifies SEC24D expression in all measured human tissues (including colon, heart, hypothalamus, kidney, liver, lung, ovary, skeletal muscle, spleen testes, and adipose tissue). Taken together, these data indicate that *Sec24d* is expressed early and broadly across tissues.

### *Sec24d* BAC Transgenes Rescue the Embryonic Lethal *Sec24^gt/gt^* Phenotype

Two independent BAC transgenes, both spanning the full *Sec24d* gene (Figure 3), exhibited rescue of the *Sec24d^gt/gt^* embryonic lethal phenotype (Table 4). *Sec24d^gt/gt^ Tg+* mice appeared healthy, and exhibited normal fertility and lifespan with no apparent abnormalities on gross autopsy. The *Sec24d^gt/gt^ Tg+* genotypes were confirmed by progeny testing (Table 5). While both BACs were able to rescue *Sec24d^gt/gt^* mice, all of the transgenes generated less than the expected number of *Sec24d^gt/gt^ Tg+* rescues. A range of penetrance was observed, both between the RP23 and RP24 (average of 58% and 37.5%, respectively) and within founder lines of individual BACs (penetrance for RP23 transgene founders range from 40–75% and for RP24 founders, from 23–58%).

**Table 4:** Rescue of *Sec24d^gt/gt^* mice by BAC transgenes.

Genotype:	*Sec24d^gt/gt^ Tg+*	other	*p-*value
**Expected Ratio with Rescue**	**14.3% (1/7)**	**85.7% (6/7)**	
Total for RP23 BAC (n = 109)	8.3% (9)	91.7% (100)	<7.3×10^−02^
RP23-677 (n = 53)	5.7% (3)	94.3% (50)	<7.3 ×10^−02^
RP23-686 (n = 56)	10.7% (6)	89.3% (50)	<4.5×10^−1^
Total for RP24 BAC (n = 243)	5.3% (13)	94.7% (230)	<6.9×10^−05^
RP24-122 (n = 61)	3.3% (2)	96.7% (59)	<1.5×10^−02^
RP24-139 (n = 85)	3.5% (3)	96.5% (82)	<4.6×10^−03^
RP24-157 (n = 97)	8.3% (8)	91.8% (89)	<9.0×10^−02^
**Total (n = 352)**	**6.25% (22)**	**93.75% (330)**	**<1.7×10^−05^**

Genotypes indicated are of 2 week old progeny resulting from a cross between *Sec24d^+/gt^ Tg+* mice and *Sec24d^+/gt^* mice.

**Table 5:** Progeny testing of *Sec24d^gt/gt^ Tg+* mice.

Genotype:	*Sec24d^+/+^ Sec24d^+/+^ Tg+*	other	
**Expected Ratio if parent was Sec24d^+/gt^, BAC-Tg+**	**28.6% (2/7)**	**71.4% (5/7)**	***p*** **-value**
Total for RP23 BAC (n = 47)	0%	100% (47)	<1.5×10^−05^
RP23-677 (n = 53)	0%	100% (47)	<1.5×10^−05^
Total for RP24 BAC (n = 83)	0%	100% (83)	<8.4×10^−09^
RP24-139 (n = 53)	0%	100% (53)	<4.2×10^−06^
RP24-157 (n = 30)	0%	100% (30)	<5.4×10^−04^
**Total (n = 130)**	0%	100% (130)	<5.6×10^−13^

Tested mice were crossed with *Sec24d^+/gt^* mice, and progeny were genotyped. The presence of any *Sec24d^+/+^* mice would indicate that the test parent was heterozygous for the gene trap allele. P-values are calculated for the observed genotypes compared to the expected if the tested transgenic mouse was *Sec24d^+/gt^* rather than *Sec24d^gt/gt^*.

### A Hypomorphic *Sec24^gt2^* Allele Supports Development to Mid-embryogenesis

**Figure 6:**
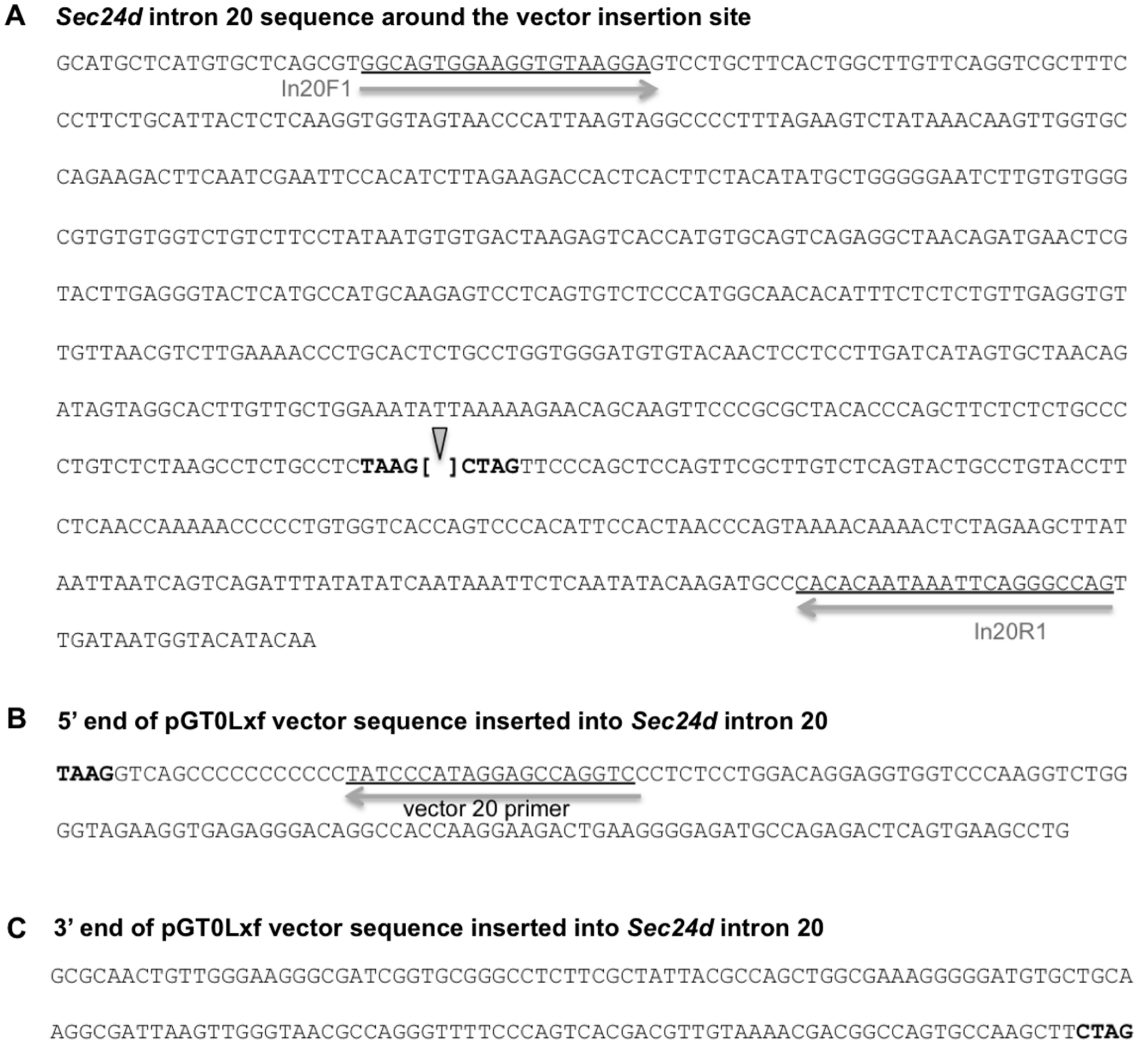
Sec24d^gt2^ allele sequence. (A) Sequence of the Sec24d^gt2^ gene trap insertion site in intron 20 of the *Sec24d* gene. The arrowhead indicates the site of the gene trap vector pGT0LxF insertion. Intron 20 sequences flanking the insertion site are in bold. The locations of genotyping primer sequences are underlined. (B) 5′ and (C) 3′ ends of the vector sequence inserted into intron 20. The position of primer V20 within the 5′ vector sequence is indicated, and flanking intron 20 sequences are in bold.

A second *Sec24d* null mouse line (*Sec24d^gt2^)* was generated from ES cells derived from an independent gene trap insertion and mapped to position 639 of intron 20 in Sec24d^gt2^ (GenBank accession number KC763190) (Figures 2, 6). The fusion transcript encoded by Sec24d^gt2^ contains SEC24D exons 1 through 20 (encoding the first 872 amino acids out of 1032) fused to the β-geo selection cassette. *Sec24d^+/gt2^* mice, like the *Sec24d^+/gt^* mice, are healthy and exhibit no apparent abnormalities upon standard autopsy examination. Intercrosses also yielded no *Sec24d^gt2/gt2^* pups at weaning (Table 6), confirming the embryonic lethality of SEC24D deficiency. In contrast to the *Sec24d^gt^* allele, analysis at both the blastocyst-stage and E10.5–11.5 identified *Sec24d^gt2/gt2^* embryos in the expected Mendelian ratio, though no *Sec24d^gt2/gt2^* embryos were observed beyond E13.5. The *Sec24d^gt2/gt2^* E10.5 embryos appeared grossly and histologically indistinguishable from their wild-type and heterozygous littermates, with visible heartbeats just prior to dissection. RT-PCR of total RNA prepared from *Sec24d^gt2/gt2^* embryos at E10.5 detected a low level of residual normal splicing around the gene trap, though quantitative analysis by PCR and Southern blotting suggests that the level of this residual full-length transcript in *Sec24d^gt2/gt2^* mice is less than 0.1% of the wild-type allele.

**Table 6:** Results of *Sec24d^+/gt2^* intercrosses, and backcrosses to *Sec24d^+/+^* mice.

*Sec24d* genotype:	+/+	+/gt2	gt2/gt2	*p-*value
***Sec24d^+/gt2^*** **×** ***Sec24d^+/gt2^*** ** expected:**	**25%**	**50%**	**25%**	
Progeny at weaning (n = 88)	34.1% (30)	65.9% (58)	0	<6.1×10^−08^
E13.5 to E18.5 (n = 29)	37.9% (11)	62.1% (18)	0	<1.9×10^−03^
E10.5 to E11.5 (n = 105)	24.8% (26)	56.2% (59)	19% (20)	<1.6×10^−01^
Blastocyst (n = 99)	26.3% (26)	47.4% (47)	26.3% (26)	<7.8×10^−01^
***Sec24d^+/gt2^***×***Sec24d^+/+^*** **expected:**	**50%**	**50%**	**–**	
Backcrosses at N2 to N12 (n = 367)	56% (207)	44% (160)	–	>1.0×10^−02^

## Discussion

Our data demonstrate that SEC24D is absolutely required for early embryonic development in the mouse, with complete deficiency resulting in uniform loss prior to the blastocyst stage. Low levels of SEC24D expression (< 0.1% of wild-type) are sufficient to support development through mid-embryogenesis, though incompatible with survival to term, whereas SEC24D haploinsufficiency results in no apparent phenotypic abnormalities. The lack of a heterozygous phenotype, together with transgenic rescue of the homozygous null phenotype, excludes a contribution from a dominant negative effect of the truncated SEC24D fusion protein to the embryonic lethality. The transgenic rescue also excludes a passenger gene effect at a nearby locus related to the gene targeting [35]. The transgenic rescue also demonstrates that the critical *cis*-regulatory sequences required for SEC24D expression are contained within the ∼140 Kb genomic segment shared by the 2 BACs used in these experiments.

The reduction in null embryos as early as the 8-cell stage (Table 2) suggests that residual maternal SEC24D is insufficient to maintain normal cellular function beyond the first few cell divisions. These data suggest a role for an essential secretory cargo in the early embryo that is specifically dependent on SEC24D for transport from the ER. Alternatively, SEC24D could be the major or only *Sec24* paralog expressed at this early developmental stage. In either case, the low level of normal *Sec24d* mRNA (<0.1%) resulting from residual splicing around the hypomorphic *Sec24d^gt2^* gene trap allele appears to be sufficient to support development past this stage, though the corresponding level of wild-type SEC24D protein was not directly measured. Also, a higher level of residual splicing in the early embryo, or partial function of the SEC24D β-gal fusion protein cannot be excluded.

The early essential role for SEC24D contrasts with the isolated neural tube developmental defect resulting from SEC24B deficiency in the mouse [19]. These results are surprising, in light of the observation of a higher extent of sequence identity for SEC24A and B to the essential yeast Sec24p protein than the SEC24C and D vertebrate paralogs, which are closer to the nonessential yeast genes Lst1p and Iss1p. The profound dependence of the early mammalian embryo on SEC24D was also unexpected, given the much milder phenotype observed in SEC24D deficient zebrafish. The latter animals exhibit craniofacial dysmorphology, thought to result from a specific defect in collagen secretion from chondrocytes [24], but otherwise develop normally. Variances in the level of maternal mRNA deposition between mice and zebrafish is not a likely explanation for these differences given the lengthy embryonic survival of the zebrafish compared to the mouse [24]. Rather, these observations suggest that the specific functions of the vertebrate SEC24s, mediated either through unique cargo selectivity or tissue-specific expression programs, may have shifted over evolutionary time. Consistent with this notion, the phenotypes of SEC23B deficiency differ markedly between humans, mice and zebrafish [21], [22], [23], [36].

The initial F_1_ and N_5_ intercrosses of *Sec24^gt/+^* heterozygous mice revealed a puzzling excess of heterozygotes compared to wild-type offspring, significantly exceeding the expected 2∶1 ratio (Table 2, *p*<0.0094). This apparent selective advantage to the *Sec24^gt/+^* heterozygote was no longer evident after further backcrossing into C57BL/6J, with genotyping of 634 backcross animals no longer showing an imbalance between the *Sec24^gt/+^* and *Sec24^+/+^* genotypes. Intercrosses of the second gene trap allele (*Sec24^gt2^*) also failed to confirm an excess of heterozygous offspring (Table 6). Taken together, these data suggest the presence of an incidental “passenger” gene mutation at a locus tightly linked to the initial *Sec24^gt^* allele [35], which was eventually lost as a result of backcrossing to C57BL/6J.

The early embryonic lethality observed in SEC24D-deficient mice is consistent with the absence of a previously identified human SEC24D phenotype, though human deficiencies have also not yet been identified for any of the other Sec24 paralogs. However, the specific phenotypes of human mutations at *SEC23A* and *SEC23B* suggest the possibility of unique disorders associated with more subtle SEC24D mutations. Only 2 SEC23A-deficient pedigrees have been identified, each carrying unique missense mutations, likely associated with a partial change/loss of function. Similarly, though many different *SEC23B* mutations have been identified in patients with CDAII, no patients have yet been identified who are homozygous or compound heterozygous for 2 null mutations, suggesting that complete SEC23B deficiency might also result in early lethality. The diverse phenotypes of humans, mice, and zebrafish with mutations in genes encoding components of the COPII coat suggests a complex balance of function among the multiple paralogous genes in each family. The availability of genetic models for deficiency in COPII component genes should enable future studies of COPII function and cargo selection *in vivo*.
